# Reduced diaphragmatic function during term labor and its association with second stage of labor: an intrapartum ultrasound study

**DOI:** 10.3389/fphys.2025.1713065

**Published:** 2026-01-12

**Authors:** Chunfeng Liu, Shijie Zhang, Huilan Hong, Yongjian Chen, Guorong Lyu

**Affiliations:** 1 Department of Obstetrics and Gynecology, Second Affiliated Hospital of Fujian Medical University, Quanzhou, Fujian, China; 2 Department of Ultrasound, Second Affiliated Hospital of Fujian Medical University, Quanzhou, Fujian, China; 3 Department of Clinical Medicine, Quanzhou Medical College, Quanzhou, Fujian, China

**Keywords:** diaphragm, intrapartum ultrasound, labor physiology, respiratory physiology, second stage of labor

## Abstract

**Background:**

To investigate differences in diaphragmatic function between women undergoing term labor and healthy non-pregnant women, and to analyze the correlation between diaphragmatic function and duration of the second stage of labor. Key obstetric factors such as pre-labor BMI, estimated fetal weight, parity, oxytocin and epidural use were considered as potential confounders.

**Methods:**

This prospective study was conducted at a tertiary perinatal center and included 94 women with term, singleton, cephalic pregnancies who underwent spontaneous vaginal delivery between December 2024 and April 2025. Diaphragmatic excursion and thickness were measured under different states during labor. A control group of 31 healthy non-pregnant women, matched for age, height, weight, and BMI, was also recruited. Differences in diaphragmatic excursion, thickness, and thickening ratio between the two groups were compared. Associations between diaphragmatic parameters and the duration of the second stage of labor were analyzed after adjusting the covariates selected by LASSO. It should be noted that over half of the controls had prior childbirths, which may modify baseline diaphragmatic morphology and introduce residual confounding.

**Results:**

In the labor group, tidal excursion and deep breath excursion were significantly lower than in controls (Effect size (95% CI) = −0.31 (−0.46, −0.16), *P* < 0.001, and −0.45 (−0.58, −0.30), *P* < 0.001). Deep inspiratory and Valsalva thickness were significantly lower in the labor group (−0.29 (−0.44, −0.11), *P* = 0.001, and −0.26 (−0.41, −0.10), *P* = 0.003). The deep breath and Valsalva thickness fractions were also reduced (−0.20 (−0.36, −0.04), *P* = 0.026, and −0.19 (−0.36, −0.01), *P* = 0.037). After LASSO regression, covariates including pre-labor BMI, parity and epidural use were selected. After adjusting for covariates (pre-labor BMI, parity and epidural), tidal expiratory and inspiratory thickness were positively correlated with the duration of the second stage of labor (β (95% CI) = 0.229 (3.286, 39.628), *P* = 0.021, and 0.201 (0.917, 32.855), *P* = 0.0380, whereas the deep breath thickness fraction was negatively correlated (−0.187 (−0.463, −0.005), *P* = 0.046).

**Conclusion:**

Women in term labor exhibited reduced diaphragmatic excursion and thinner diaphragmatic thickness under functional conditions compared with non-pregnant women. Observed associations indicated that tidal inspiratory thickness and deep breath thickness fraction were related to the duration of the second stage of labor. It should be noted that over half of the control participants had prior childbirths, which may influence baseline diaphragmatic morphology and introduce residual confounding. Given the observational design, causal inferences cannot be drawn.

## 1 Background

Inadequate maternal expulsive force during labor can lead to prolonged labor, increasing the risk of fetal distress, infection, soft tissue injury, and postpartum hemorrhage (Kissler et al., 2020; Cheng et al., 2007; Okeahialam et al., 2024). Maternal expulsive force consists of both uterine contractility and maternal pushing (Liao et al., 2005; Grimm, 2016). Maternal pushing (Valsalva maneuver) can significantly increase intrauterine pressure to promote fetal expulsion (Grimm, 2021). During the second stage of labor, ineffective maternal pushing efforts not only result in physiological and psychological fatigue but also prolong labor, thereby elevating the rates of operative deliveries and neonatal morbidity, as well as the risk of postpartum pelvic floor injury (Okeahialam et al., 2024; Niemczyk et al., 2022; LeFevre et al., 2021).

The coordination among the diaphragm, abdominal muscles, and pelvic floor forms a unified pressure-generation system that is important to the biomechanics of second stage of labor (Buhimschi et al., 2002; Zachovajeviene et al., 2019). During the effective maternal expulsive effort, the diaphragm drives downward displacement that increases transdiaphragmatic pressure, the abdominal muscles constrict inward to further elevate intra-abdominal pressure, and the levator ani complex regulates the direction of pressure transmission by modulating pelvic outlet resistance (Li et al., 2011; Demaria et al., 2005). Previous studies describe these three components as an integrated “diaphragm–abdominal–pelvic floor synergy,” in which coordinated contraction is required to translate muscular force into intra-abdominal pressure that augments uterine forces and promotes fetal descent (Grimm, 2021; Çiçek et al., 2024; Çiçek et al., 2023). Pregnancy-related physiological adaptations—including diaphragmatic elevation caused by uterine enlargement, reduced diaphragmatic excursion, increased chest wall circumference, and decreased thoracic compliance—may impair this synergy by limiting pressure generation or changing its directionality (Çiçek et al., 2024; Çiçek et al., 2023; Tan and Tan, 2013; Nason et al., 2012). These changes not only suggest that the diaphragmatic contribution to intra-abdominal pressure may differ between parturients and healthy non-pregnant women, but also provide a biomechanic rationale for why impaired diaphragmatic function could influence the effectiveness of expulsive efforts and thereby affect the second stage of labor.

Diaphragmatic ultrasound is a non-invasive technique that allows real-time visualization of diaphragmatic displacement and thickening (Truong et al., 2023; Sferrazza Papa et al., 2016). Studies have shown that it is a feasible and accurate tool for assessing diaphragmatic anatomy and respiratory physiology, commonly used in the diagnosis of neuromuscular disorders, respiratory diseases (such as COPD), and respiratory impairment caused by stroke (Sferrazza Papa et al., 2016; Moury et al., 2024; Kharaji et al., 2023; Baria et al., 2014; Chen et al., 2022). Additionally, diaphragmatic ultrasound can guide the placement of electromyographic electrodes (to avoid pneumothorax) and provide valuable information for managing critically ill patients, such as ventilator-associated diaphragmatic dysfunction (Sferrazza Papa et al., 2016). Common diaphragmatic ultrasound parameters include diaphragmatic excursion, diaphragmatic thickness, and diaphragmatic thickening fraction (Haaksma et al., 2022). These parameters assist in the quantitative assessment of diaphragmatic contraction during inspiration and the extent of diaphragmatic descent, thereby evaluating diaphragmatic function. Due to its non-invasive and radiation-free nature, this technique is suitable for use in parturients (Tomà et al., 2019).

Given the critical role of the diaphragm in the coordination and the physiological adaptations during labor, we hypothesized that compared with healthy non-pregnant controls, term parturients exhibit reduced diaphragmatic performance; additionally, diaphragmatic functional parameters may be associated with the duration of the second stage of labor. These potential associations are exploratory and do not imply causality. Currently, no studies have utilized diaphragmatic ultrasound to assess diaphragmatic function during labor in parturients. Therefore, this study aims to explore the differences in diaphragmatic function between women undergoing term labor and healthy non-pregnant women and to analyze the correlation between diaphragmatic function and duration of the second stage of labor, thereby establishing a foundation for future research.

## 2 Materials and methods

This study is a prospective observational study conducted at a single tertiary perinatal center. Given the exploratory nature of this research and the lack of prior effect-size estimates, we pre-specified a convenience-based, consecutive enrollment strategy. Parturients who delivered at this center from December 2024 to April 2025 were therefore recruited consecutively. The inclusion criteria were as follows: singleton term pregnancy, fetal head presentation, spontaneous vaginal delivery, age ≥18 years, and the ability to understand and provide informed consent. The exclusion criteria included diseases that may affect diaphragmatic measurements (e.g., pneumonia, pleuritis, peritonitis, chronic respiratory diseases), poor image quality (e.g., due to obesity), inability to cooperate in performing an effective Valsalva maneuver, and substance use disorders such as smoking, alcohol, or drug addiction. Perinatal management was conducted in accordance with clinical guidelines (Chinese Maternal and Child Health Care Association, 2020; Chinese Medical Association et al., 2020). A control group of healthy non-pregnant female volunteers was also recruited, with age, height, weight, and BMI matched to those of the study group (comparing pre-pregnancy weight and BMI). This study adhered to the Strobe (Strengthening the Reporting of Observational Studies in Epidemiology) guidelines. The study was approved by the Medical Research Ethics Committee of the Second Affiliated Hospital of Fujian Medical University in September 2024 (approval number: 2024FYFELLSZ531), and all participating women provided written informed consent.

### 2.1 Demographic and obstetric data

Demographic and obstetric data were collected from medical records, including maternal age, height, weight, pre-pregnancy weight, gestational age, estimated fetal weight (based on the most recent ultrasound), parity at enrollment, use of oxytocin, use of epidural anesthesia, duration of the second stage of labor (from full cervical dilation to fetal expulsion), and delivery outcomes (spontaneous vaginal delivery or operative delivery).

### 2.2 Ultrasound examination

Ultrasound examinations were performed using the Mindray Z6 ultrasound diagnostic system, equipped with a 3C5P convex array probe, a 2P2P phased array probe, and a 7L4P linear array probe. The examination was conducted when the parturient’s cervix was fully dilated and ready for active pushing. The parturient was positioned supine on the delivery table with her hips and knees flexed and externally rotated. Prior to the examination, the parturient was adequately instructed on how to perform the Valsalva maneuver.

Diaphragmatic excursion was measured using a low-frequency convex array probe or a phased array probe (2–5 MHz) in M-mode. The probe was placed between the right midclavicular line and the right anterior axillary line, with the probe handle gently pressed to align the ultrasound beam as closely as possible with the diaphragm dome. The maximum depth was adjusted to ensure the entire diaphragmatic motion cycle was visible on the screen. Measurements were taken during both tidal breathing and deep breathing. When the diaphragm dome was not clearly visualized, organ displacement was used as an alternative. Each measurement was repeated at the same location to ensure consistency and comparability of the data.

Diaphragmatic thickness was measured using a linear array probe (7–12 MHz) in B-mode. The probe was placed at the mid-axillary line or slightly anteriorly, approximately between the 6th and 10th ribs, ensuring that the lung image appeared only at the edge of the screen. The probe was positioned perpendicular to the chest wall to ensure visualization of the pleura, peritoneum, and diaphragm muscle layers. The gain was adjusted appropriately to ensure accurate identification of the diaphragm boundaries. The outer edge of the calipers was placed at the inner edge of the pleura and peritoneum lines to precisely measure the actual diaphragmatic thickness. Measurements were taken at the end of tidal expiration, tidal inspiration, deep inspiration, and during the Valsalva maneuver. The diaphragmatic thickening fraction was calculated using the formula: (inspiratory thickness - expiratory thickness)/expiratory thickness × 100%.

Considering that maternal expulsive efforts during labor occur in the Valsalva state, the diaphragmatic thickening fraction measured during the Valsalva maneuver was defined as the primary outcome variable. The remaining variables—including diaphragmatic excursion during tidal and deep breathing, diaphragmatic thickness at end-tidal expiration, end-tidal inspiration, deep inspiration, and Valsalva states, as well as the diaphragmatic thickening fraction during deep inspiration—were defined as secondary outcome variables.

Given that diaphragmatic thickness measurement is a complex skill with a steep learning curve, strict quality control measures were implemented. All researchers underwent standardized training, which included diaphragmatic anatomy, anatomical landmark identification, supervised practice, and practical assessments. Each researcher completed at least 40 examinations (with at least 20 performed under direct or indirect supervision of an experienced ultrasound physician) to ensure they could reliably and independently apply these skills in routine clinical practice. Additionally, to further improve measurement accuracy, all data were reviewed by two independent researchers (C.L. and S.Z.), with any discrepancies resolved by a third-party expert (G.L.). Although inter-observer and intra-observer reliability testing was not performed in this study, all measurements were acquired following standardized protocols and were conducted by experienced operators. Previous studies have demonstrated good to excellent reproducibility for ultrasound assessment of diaphragmatic excursion and thickness, with intraclass correlation coefficients typically ranging from 0.86 to 0.97 (Kharaji et al., 2023).

### 2.3 Confounders of diaphragm measurement

Standardized breathing instructions and Valsalva coaching were provided by trained midwives. Women were allowed to adopt free birthing positions during labor. Pre-labor BMI, estimated fetal weight, parity, oxytocin and epidural use were included as potential factors associated with diaphragmatic function and labor progression (Çiçek et al., 2024; Shao et al., 2025; Callahan et al., 2023). It should be noted that maternal physical conditioning, breathing strategies, and pelvic floor function may also influence diaphragmatic performance, but these variables were not measured in this study and may have introduced residual confounding. In addition, over half of the control participants had previously delivered, whiles most women in the delivery group were nulliparous. This imbalance may influence baseline diaphragmatic thickness and excursion, and represents an inevitable source of residual confounding.

### 2.4 Statistical analysis

Continuous variables were tested for normality using Kolmogorov-Smirnov and Shapiro-Wilk test (Supplementary Table 1). As most variables did not follow a normal distribution and the unequal sample sizes between groups, continuous data were presented as medians with interquartile ranges. For between-group comparisons, the Mann-Whitney U test was used for continuous variables. Categorical variables were summarized as frequencies and percentages, and compared by the chi-square test or Fisher’s exact test as appropriate. For within-group comparisons, the Friedman test was employed for continuous variables, with *post hoc* pairwise comparisons conducted using the q test and adjusted with the Holm correction. Multivariable linear regression analyses were performed to explore the association between diaphragmatic function and duration of the second stage of labor. Covariates in the regression models were selected based on prior literature and physiological plausibility, including pre-labor BMI, parity, oxytocin and epidural analgesia, all of which are regarded as influential factors related to diaphragmatic function and the labor process (Çiçek et al., 2024; Shao et al., 2025; Callahan et al., 2023). LASSO regression was used for screening covariates. Statistical analyses were conducted using R software (version 4.1.3; R Core Team, Vienna, Austria). A two-sided P-value ≤0.05 was considered statistically significant. This study is exploratory and hypothesis generating. Because no prior effect-size estimates for intrapartum diaphragmatic ultrasound were available, this study was designed as an exploratory observational project using a convenience sample. The sample size was determined *a priori* by the fixed 5-month recruitment window (December 2024–April 2025), and all eligible parturients during this period were consecutively enrolled. Therefore, the resulting sample should be interpreted as hypothesis-generating rather than powered for definitive inference. A *post hoc* power analysis was conducted based on the final dataset. Using the Mann–Whitney U test to compare the two groups, the parturient group (n = 94) had a median of 70.71 with an IQR of 44.39, and the control group (n = 31) had a median of 91.38 with an IQR of 70.16. Assuming α = 0.05 and a target power of 0.80, the standard deviations were estimated from the IQR values (SD ≈ IQR/1.35), yielding SDs of 32.88 and 51.97, respectively. Cohen’s d was calculated as approximately 0.55 and converted to a non-parametric effect size r of about 0.26. Based on these parameters, G*Power 3.1 indicated an achieved power of 0.92, exceeding the target threshold and suggesting adequate sensitivity (with an 8% Type II error risk) to detect a moderate between-group difference.

## 3 Results

### 3.1 Demographic and obstetric characteristics

A total of 94 parturients and 31 healthy non-pregnant women were ultimately enrolled. The labor group and the control group were comparable in terms of age, height, weight, BMI and parity (Table 1). In the labor group, the majority were nulliparous women 56 (59.57%), approximately half underwent labor induction 47 (50.00%), and most received epidural analgesia 72 (76.60%). The majority of parturients achieved spontaneous vaginal delivery 84 (89.36%).

**TABLE 1:** Demographic and obstetric data of parturients and controls.

Variable	Parturition (n = 94)	Control (n = 31)	P value	Effect size (95% CI)
Age, year	30.00 (7.75)	32.00 (8.00)	0.134	−0.13 (−0.32, 0.06)
Height, cm	160.00 (8.00)	160.00 (4.00)	0.491	−0.06 (−0.21, 0.09)
Weight, kg	55.00 (12.50)	58.00 (8.00)	0.210	−0.11 (−0.26, 0.05)
BMI, kg/m^2^	21.64 (3.68)	22.58 (3.42)	0.365	−0.08 (−0.23, 0.08)
Pregnant weight, kg	69.00 (11.00)	—	—	—
Pregnant BMI, kg/m^2^	27.20 (4.28)	—	—	—
EFW, g	3400.00 (372.00)	—	—	—
Gestational age, days	277.00 (9.75)	—	—	—
Primiparous	56 (59.57%)	14 (45.16%)	0.233	0.13 (0.01, 0.30)
Oxytocin	47 (50.00%)	—	—	—
Epidural anesthesia	72 (76.60%)	—	—	—
SSL, min	32.50 (57.00)	—	—	—
SVD	84 (89.36%)	—	—	

Values reported as median (interquartile range) or frequency (%) as appropriate.

Mann Whitney U test was used for continuous variables, and chi square test or Fisher’s exact test was used for categorical variables.

SSL, Second Stage of Labor; SVD, spontaneous vaginal delivery.

### 3.2 Comparison between maternal and control groups

The diaphragm excursion during both tidal breathing and deep breathing was significantly lower in the labor group compared to the control group (both P < 0.001). The diaphragm thickness at deep inspiration and during the Valsalva maneuver was also significantly thinner in the labor group than in the control group (P = 0.001, P = 0.003) (Table 2). Additionally, the diaphragm thickening fraction during deep breathing and the Valsalva maneuver was significantly lower in the labor group (P = 0.026, P = 0.037).

**TABLE 2:** Comparison of diaphragmatic function between parturients and controls.

Variable	Parturition (n = 94)	Control (n = 31)	Test statistic	P value	Effect size (95% CI)
TE, cm	1.73 (0.69)	2.08 (0.66)	2073	**<0.001**	−0.31 (−0.46, −0.16)
DE, cm	4.82 (1.38)	6.25 (1.43)	2344	**<0.001**	−0.45 (−0.58, −0.30)
TET, cm	0.16 (0.06)	0.16 (0.10)	1603.5	0.402	−0.07 (−0.26, 0.10)
TIT, cm	0.18 (0.06)	0.18 (0.11)	1628	0.329	−0.09 (−0.27, 0.11)
DIT, cm	0.26 (0.12)	0.30 (0.14)	2015	**0.001**	−0.29 (−0.44, −0.11)
VT, cm	0.28 (0.16)	0.34 (0.15)	1970.5	**0.003**	−0.26 (−0.41, −0.10)
TTF, %	15.79 (10.36)	11.54 (7.78)	1123.5	0.057	0.17 (−0.01, 0.35)
DTF, %	59.94 (42.95)	70.00 (55.71)	1846.5	**0.026**	−0.20 (−0.36, −0.04)
VTF, %	70.71 (44.39)	91.38 (70.16)	1822.5	**0.037**	−0.19 (−0.36, −0.01)

Values reported as median (interquartile range) and Mann Whitney U test was used.

TE, Tidal excursion; DE, Deep breath excursion; TET, Tidal expiratory thickness; TIT, Tidal inspiratory thickness; DIT, Deep inspiratory thickness; VT, Valsalva thickness; TTF, Tidal thickness fraction; DTF, Deep breath thickness fraction; VTF, valsalva thickness fraction. Bold values indicate statistical significance (P < 0.05).

**FIGURE 1:**
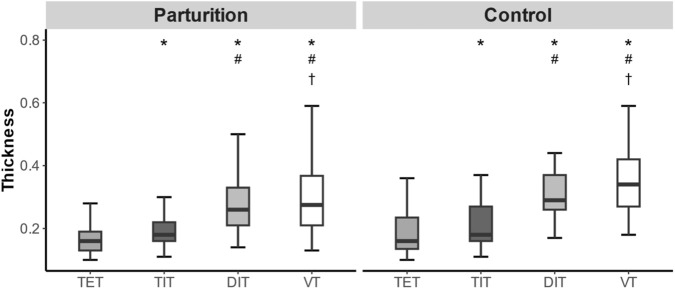
Box plot of parturients and controls. Friedman test was used for intra group analysis. Afterwards, a q-test was used for pairwise comparison and Holm correction was performed. * Compared with TET, the difference is statistically significant, and after q test with Holm correction, P < 0.05. # Compared with TIT, the difference is statistically significant, and after q test with Holm correction, P < 0.05. † Compared with DIT, the difference is statistically significant, and after q test with Holm correction, P < 0.05. TET, Tidal expiratory thickness. TIT, Tidal inspiratory thickness. DIT, Deep inspiratory thickness. VT, Valsalva thickness.

Compared with tidal expiratory thickness, the diaphragm thickness at tidal inspiratory, deep inspiratory, and Valsalva states was significantly greater in both groups (all P < 0.001). Compared with tidal inspiratory thickness, the diaphragm thickness at deep inspiration and during the Valsalva maneuver was also significantly greater in both groups (all P < 0.001). Furthermore, compared with deep inspiratory thickness, the diaphragm thickness during the Valsalva maneuver was significantly greater in both groups (P = 0.005, P = 0.032) (Figure 1).

### 3.3 Correlation analysis

The pregnant BMI, parity and epidural use were selected by LASSO regression analysis as covariates. After adjusting for covariates, correlation analysis revealed that diaphragm thickness at tidal expiration and tidal inspiration had a significant positive association with the duration of the second stage of labor, whereas diaphragm thickening fraction during deep breathing was significantly negatively associated with the duration of the second stage (Table 3). However, despite model simplification and LASSO-based covariate selection, several adjusted coefficients demonstrated wide confidence intervals, indicating residual model instability and limited precision of the estimated associations.

**TABLE 3:** Correlation analysis between diaphragm thickness and second stage of labor.

Variable	Non standardized β	Standardizedβ (95% CI)	P
TE	0.175	0.019 (−1.539, 1.889)	0.840
DE	0.052	0.014 (−0.644, 0.748)	0.882
TET	21.457	0.229 (3.286, 39.628)	0.021
TIT	16.886	0.201 (0.917, 32.855)	0.038
DIT	2.302	0.038 (−9.703, 14.306)	0.704
VT	5.044	0.109 (−4.479, 14.566)	0.295
TTF	−0.308	−0.071 (−1.157, 0.54)	0.472
DTF	−0.234	−0.187 (−0.463, −0.005)	0.046
VTF	−0.104	−0.083 (−0.34, 0.132)	0.382

TE, Tidal excursion; DE, Deep breath excursion; TET, Tidal expiratory thickness; TIT, Tidal inspiratory thickness; DIT, Deep inspiratory thickness; VT, Valsalva thickness; TTF, Tidal thickness fraction; DTF, Deep breath thickness fraction; VTF, Valsalva thickness fraction.

The sensitivity analysis shows that TET and TIT were generally positively correlated with the second stage of labor, but the effect weakened after removing outliers. Stratified by parity, this effect was mainly concentrated in primiparous women, and interaction analysis suggested that parity may be a modifying factor of the effect. This can indicate that the results have a certain degree of robustness, but caution should still be exercised when interpreting them, especially when there is no significant effect in multiparous women (Table 4).

**TABLE 4:** Sensitivity analysis.

Variable	Analysis_Type	Standardizedβ (95% CI)	p
TET	Remove outliers	0.143 (−6.949, 36.868)	0.178
TIT	Remove outliers	0.124 (−7.285, 30.298)	0.226
TET	Parity = 0	0.362 (5.196, 62.413)	0.022
TET	Parity >0	−0.03 (−17.131, 14.459)	0.864
TIT	Parity = 0	0.333 (2.218, 53.879)	0.034
TIT	Parity >0	−0.065 (−16.107, 11.012)	0.704
TET	Effect modification: TET	0.027 (−25.872, 30.858)	0.862
TET	Effect modification: TET × Primiparous	0.6 (−5.543, 63.566)	0.099
TIT	Effect modification: TIT	0.006 (−24.276, 25.212)	0.970
TIT	Effect modification: TIT × Primiparous	0.616 (−4.907, 57.049)	0.098

TET, Tidal expiratory thickness; TIT, tidal inspiratory thickness.

### 3.4 Subgroups analysis

Subgroup analyses of primiparous vs. multiparous (56 vs. 38), epidural analgesia vs. no epidural (72 vs. 22), and induced vs. spontaneous labor (47 vs. 47) are shown in Supplementary Table 2. Due to the limited sample sizes in some subgroups, these analyses were underpowered and should be considered exploratory; the findings are preliminary and cannot be interpreted as confirmatory evidence.

## 4 Discussion

The results of this study demonstrated that term parturients exhibited reduced diaphragm excursion and thinner diaphragm thickness at deep inspiration and during the Valsalva maneuver compared to healthy non-pregnant women. These findings suggest that maternal diaphragmatic function may be associated with various physiological and mechanical changes associated with pregnancy. During pregnancy, uterine enlargement and increased intra-abdominal pressure elevate the diaphragm by approximately 4 cm, restricting its range of motion and resulting in more shallow, rapid breathing, thereby reducing diaphragm excursion (Tan and Tan, 2013; Hegewald and Crapo, 2011). In addition, hormonal changes during pregnacy, such as increased levels of estrogen, progesterone, and relaxin, may impair diaphragmatic contractility and alter muscle mass, contributing to a thinner diaphragm during contraction (Tan and Tan, 2013). Previous research (Çiçek et al., 2024) also reported a significant decline in diaphragmatic thickness during late pregnancy compared with mid-pregnancy, further supporting these findings.

Furthermore, our study found that diaphragm thickening fraction during deep breathing and the Valsalva maneuver was lower in parturients than in healthy controls. Given the increased intra-abdominal pressure during these maneuvers, the diaphragm is required to generate greater force, and its thickening fraction may be limited due to pregnancy-induced physiological muscle changes. These observations may provide insights into understanding respiratory function alterations during labor. The subgroup findings, although potentially informative, are exploratory due to small sample sizes. They should be interpreted with caution and are not confirmatory and require validation in larger studies.

The correlation analysis in this study indicated that greater diaphragm thickness during tidal breathing was associated with a prolonged second stage of labor, whereas a higher diaphragm thickening fraction during deep breathing was linked to a shorter second stage. The diaphragm, as the principal respiratory muscle, also plays a critical role in modulating intra-abdominal pressure (Çiçek et al., 2024; Tan and Tan, 2013). During labor, the rhythmic contraction and relaxation of the diaphragm may synergize with uterine contractions, contributing to the progression of labor. It is worth noting that the association between increased tidal thickness and prolonged second stage in this study may be related to physiological adaptation of the diaphragm under high load during labor (McCool and Tzelepis, 2012). On the one hand, an increase in thickness during the tidal phase may indicate a compensatory baseline activation, namely, sustained muscle tension or spasmodic activity (Rives et al., 2021; Zacarias Rondinel et al., 2024). On the other hand, an increase in baseline thickness may be related to a decrease in shrinkage rate (i.e., lower thickening fraction). These explanations are all hypothetical in nature, as this study did not directly measure electromyographic or abdominal pressure parameters, but could serve as a potential avenue for understanding changes in diaphragmatic dynamics during labor. After adjusting the pregnant BMI, parity and epidural use, the increased thickness in the tidal phase was still significantly associated with the prolonged second stage. However, these adjusted associations should be interpreted with caution, as the wide confidence intervals and potential sparse-data effects suggest underlying model instability that may reflect limited subgroup sizes and predictor imbalance. Sensitivity analysis further indicates that the positive correlation between tidal thickness and the second stage of labor is generally stable, but the effect weakens after removing outliers. Stratified analysis shows that this effect mainly exists in primiparous women, and is not significant in multiparous women. Interaction analysis also suggests that parity may be an important modifying factor affecting this effect. Therefore, although the results have a certain degree of robustness, caution should still be exercised when interpreting them, especially regarding the heterogeneity that may exist in different parity populations.

Previous studies have shown that postpartum women may experience long-term adaptive changes in pelvic floor and abdominal wall muscles after previous childbirth, thereby altering the regulation of abdominal pressure (Bø et al., 2022). In addition, after the first vaginal delivery, the perineal and pelvic tissues become more compliant (Emmerson et al., 2017). In contrast, primiparous women have greater resistance to fetal head descent and birth canal dilation, and rely more on the coordinated force between the diaphragm, abdominal muscles, and pelvic floor muscles (Grimm, 2021). Therefore, the observation in this study has certain physiological rationality, but further verification is still needed in larger samples.

Interestingly, previous studies have reported that the normal threshold for diaphragm thickness in healthy individuals typically exceeds 0.2 cm (Haaksma et al., 2022). However, in the present study, the median diaphragm thickness in the healthy non-pregnant group was 0.16 cm. We believe that this phenomenon is closely related to the demographic characteristics of the control group. First, all participants in this study were from the southeastern coastal region of Asia, where the average female BMI (20–23 kg/m^2^) is significantly lower than that reported for Western populations (25–28 kg/m^2^) (NCD Risk Factor Collaboration NCD-RisC, 2016). Second, more importantly, 17 participants (54.84%) in the control group had a history of previous childbirth. Previous studies have shown that prior pregnancies may lead to long-term adaptive changes in diaphragmatic thickness or function (Çiçek et al., 2024; Kharaji et al., 2023), which means that the baseline diaphragm morphology of the control group may not be completely consistent with that of real “healthy women who have not experienced pregnancy.” This mismatch may not only result in an overall lower diaphragm thickness in the control group, but also affect the range of diaphragm excursion, thereby weakening or even masking the true differences between the delivery group and the control group. In other words, this bias may lead to underestimation of inter group differences and affect the explanatory power of differences in diaphragm function. Therefore, when interpreting inter-group comparisons, this impact should be fully recognized and considered as an important internal validity limitation of this study.

This study did not quantify maternal physical condition, breathing strategy, labor coaching, and pelvic floor status, all of which may influence diaphragmatic performance and labor progression. For example, reduced physical fitness or pelvic floor dysfunction may diminish diaphragmatic contraction efficiency, affecting intra-abdominal pressure generation and, consequently, labor progress. Variations in breathing patterns or pushing techniques may also alter diaphragmatic movement. In addition, the specific impact of free birthing positions on diaphragmatic function remains unclear. Because these unmeasured factors are closely related to diaphragmatic function, they may have introduced residual confounding, and the findings should be interpreted within this context. In summary, pregnancy-related physiological changes were potentially associated with reduced diaphragmatic function and long-term alterations in diaphragm thickness. Based on these findings, future studies could explore the potential role of diaphragmatic training during pregnancy on labor efficiency, investigate the association between diaphragmatic function during labor and second stage duration, and assess the effects of postpartum diaphragmatic exercises.

This study has several limitations. First, this study is an exploratory prospective observational study that does not include prior sample size calculations based on predefined effect sizes. Although *post hoc* power analysis has been added, it does not meet the needs of demonstrating sample sufficiency. Second, although factors such as pre-labor BMI, estimated fetal weight, parity, oxytocin and epidural use were included in the regression models, maternal physical condition, breathing strategies, labor coaching, pelvic floor function, and birthing positions were not quantified, and these may represent important confounders affecting diaphragmatic function. In addition, the regression models exhibited residual instability, including wide confidence intervals for several parameters, indicating possible sparse-data bias and reduced precision. As a result, the adjusted associations identified in this study are exploratory and require validation in larger, adequately powered cohorts. Third, this study did not evaluate inter- or intra-observer reliability. Although standardized scanning procedures and experienced sonographers were employed, diaphragm ultrasound is inherently operator-dependent. Without formal reliability testing, the extent of measurement variability cannot be quantified, which may reduce measurement precision and weaken the internal validity of the observed associations. This variability may weaken the true correlation between diaphragm parameters and second stage of labor, or lead to wider confidence intervals, so the results should be interpreted with appropriate caution. Besides, over half of the control participants had a history of childbirth, while most women in the delivery group were nulliparous (56 (59.57%) vs. 14 (45.16%)). Prior childbirth can alter baseline diaphragmatic morphology by increasing abdominal wall compliance, affecting resting diaphragm thickness, and modifying excursion patterns. This mismatch may partially account for the observed between-group differences and reduces the internal validity of the comparison. Finally, this study evaluated intrapartum diaphragmatic function at a single time point, without capturing its dynamic changes throughout labor, which may limit understanding of how it evolves as labor progresses. Future studies should consider standardizing intrapartum breathing strategies, unifying maternal position and obstetric assistance guidance, incorporating objective assessments of maternal fitness, and integrating pelvic floor ultrasound or electromyography to clarify the independent contribution of these factors to diaphragmatic function during labor.

## 5 Conclusion

Women at term showed lower diaphragmatic excursion and reduced functional thickness compared with healthy non-pregnant women, suggesting that late pregnancy and labor may be associated with altered diaphragmatic biomechanics. In addition, diaphragmatic thickness at tidal breathing and thickening fraction during deep breathing were correlated with the second stage of labor, providing preliminary evidence of potential association between diaphragmatic function and delivery progression. These findings are still exploratory and primarily hypothesis-generating. Future research should focus on clarifying potential mechanisms and improving methodological rigor through larger cohorts, repeated measurements, and longitudinal study designs to better describe temporal patterns and causal pathways.

## Data Availability

The raw data supporting the conclusions of this article will be made available by the authors, without undue reservation.
