# Supplementary material for: Reduced diaphragmatic function during term labor and its association with second stage of labor: an intrapartum ultrasound study
Source: Front Physiol. 2026 Jan 12;16:1713065. doi: 10.3389/fphys.2025.1713065 (PMC12832231; doi:10.3389/fphys.2025.1713065)
Supplement: Supplementary file 1 [file Table1.docx]

Supplementary Table1 Normality test

| Variable | Kolmogorov-Smirnov _P | Shapiro-Wilk _P |
| --- | --- | --- |
| Age | 0.169 | 0.010 |
| Height | 0.050 | 0.007 |
| Weight | 0.161 | <0.001 |
| BMI | 0.189 | <0.001 |
| Pregnant Weight | 0.262 | 0.001 |
| Pregnant BMI | 0.834 | 0.084 |
| EFW | 0.045 | <0.001 |
| Gestational Age | 0.073 | 0.003 |
| Second Stage of Labor | 0.019 | <0.001 |
| TE | 0.710 | 0.870 |
| DE | 0.555 | 0.761 |
| TET | <0.001 | <0.001 |
| TIT | <0.001 | <0.001 |
| DIT | 0.015 | <0.001 |
| VT | 0.024 | <0.001 |
| TTF | <0.001 | <0.001 |
| DTF | 0.034 | <0.001 |
| VTF | 0.035 | <0.001 |

TE, Tidal excursion. DE, Deep breath excursion. TET, Tidal expiratory thickness. TIT, Tidal inspiratory thickness. DIT, Deep inspiratory thickness. VT, Valsalva thickness. TTF, Tidal thickness fraction. DTF, Deep breath thickness fraction. VTF, Valsalva thickness fraction.
