# Supplementary material for: Reduced diaphragmatic function during term labor and its association with second stage of labor: an intrapartum ultrasound study
Source: Front Physiol. 2026 Jan 12;16:1713065. doi: 10.3389/fphys.2025.1713065 (PMC12832231; doi:10.3389/fphys.2025.1713065)
Supplement: Supplementary file 2 [file Table2.docx]

# Subgroup Analysis: nulliparous vs multiparous

| Variable | Nulliparous(n=56) | Multiparous(n=38) | P | Effect_r | CI_L | CI_U |
| --- | --- | --- | --- | --- | --- | --- |
| TE | 1.83 (0.73) | 1.64 (0.63) | 0.240 | 0.122 | -0.071 | 0.319 |
| DE | 4.86 (1.30) | 4.66 (2.07) | 0.686 | -0.042 | -0.254 | 0.166 |
| TET | 0.16 (0.06) | 0.16 (0.05) | 0.395 | 0.088 | -0.113 | 0.283 |
| TIT | 0.18 (0.06) | 0.18 (0.05) | 0.521 | 0.066 | -0.131 | 0.263 |
| DIT | 0.26 (0.13) | 0.26 (0.08) | 0.173 | 0.141 | -0.064 | 0.328 |
| VT | 0.29 (0.14) | 0.24 (0.16) | 0.075 | 0.184 | -0.011 | 0.372 |
| TTF | 15.38 (10.48) | 16.67 (9.33) | 0.720 | -0.037 | -0.242 | 0.176 |
| DTF | 59.94 (44.75) | 57.92 (41.12) | 0.683 | 0.043 | -0.158 | 0.245 |
| VTF | 79.80 (36.94) | 58.68 (51.46) | 0.070 | 0.187 | -0.035 | 0.399 |

# Subgroup Analysis: epidural vs no epidural

| Variable | Epidural(n=72) | No epidural(n=22) | P | Effect_r | CI_L | CI_U |
| --- | --- | --- | --- | --- | --- | --- |
| TE | 1.70 (0.58) | 1.81 (0.81) | 0.731 | -0.036 | -0.249 | 0.201 |
| DE | 4.66 (1.56) | 5.00 (1.33) | 0.751 | -0.033 | -0.228 | 0.162 |
| TET | 0.16 (0.04) | 0.14 (0.05) | 0.003 | 0.303 | 0.116 | 0.477 |
| TIT | 0.19 (0.06) | 0.16 (0.04) | 0.013 | 0.257 | 0.062 | 0.429 |
| DIT | 0.27 (0.12) | 0.23 (0.08) | 0.006 | 0.286 | 0.117 | 0.445 |
| VT | 0.30 (0.13) | 0.21 (0.05) | 0.001 | 0.335 | 0.149 | 0.507 |
| TTF | 15.59 (9.73) | 17.42 (11.61) | 0.119 | -0.161 | -0.350 | 0.044 |
| DTF | 58.89 (44.18) | 60.42 (31.02) | 0.827 | -0.023 | -0.225 | 0.171 |
| VTF | 76.98 (41.07) | 61.90 (45.68) | 0.195 | 0.134 | -0.092 | 0.345 |

# Subgroup Analysis: induction vs spontaneous

| Variable | Induction(n=47) | Spontaneous(n=47) | P | Effect_r | CI_L | CI_U |
| --- | --- | --- | --- | --- | --- | --- |
| TE | 1.79 (0.57) | 1.66 (0.71) | 0.847 | 0.020 | -0.175 | 0.222 |
| DE | 4.62 (1.50) | 4.84 (1.34) | 0.865 | 0.018 | -0.182 | 0.226 |
| TET | 0.17 (0.06) | 0.15 (0.04) | 0.241 | 0.121 | -0.078 | 0.319 |
| TIT | 0.19 (0.06) | 0.17 (0.04) | 0.428 | 0.082 | -0.118 | 0.286 |
| DIT | 0.27 (0.11) | 0.24 (0.12) | 0.173 | 0.141 | -0.051 | 0.343 |
| VT | 0.31 (0.14) | 0.25 (0.13) | 0.124 | 0.159 | -0.043 | 0.367 |
| TTF | 15.38 (9.57) | 16.67 (8.56) | 0.223 | -0.126 | -0.325 | 0.073 |
| DTF | 66.67 (33.17) | 53.33 (52.45) | 0.228 | 0.125 | -0.077 | 0.324 |
| VTF | 81.82 (36.76) | 61.54 (46.70) | 0.077 | 0.183 | -0.013 | 0.387 |
